# Extracellular vesicles in bone: “dogrobbers” in the “eternal battle field”

**DOI:** 10.1186/s12964-019-0319-5

**Published:** 2019-01-18

**Authors:** Shi-Cong Tao, Shang-Chun Guo

**Affiliations:** 1 Department of Orthopaedic Surgery, Shanghai Jiao Tong University Affiliated Sixth People’s Hospital, 600 Yishan Road, Shanghai, 200233 China; 2 Institute of Microsurgery on Extremities, Shanghai Jiao Tong University Affiliated Sixth People’s Hospital, 600 Yishan Road, Shanghai, 200233 China

**Keywords:** Extracellular vesicles, Bone, Exosomes, Signalling pathways, Intercellular communications

## Abstract

Throughout human life, bone is constantly in a delicate dynamic equilibrium of synthesis and resorption, hosting finely-tuned bone mineral metabolic processes for bone homeostasis by collaboration or symphony among several cell types including osteoclasts (OCs), osteoblasts (OBs), osteocytes (OYs), vascular endothelial cells (ECs) and their precursors. Beyond these connections, a substantial level of communication seems to occur between bone and other tissues, and together, they form an organic unit linked to human health and disease. However, the current hypothesis, which includes growth factors, hormones and specific protein secretion, incompletely explains the close connections among bone cells or between bone and other tissues. Extracellular vesicles (EVs) are widely-distributed membrane structures consisting of lipid bilayers, membrane proteins and intravesicular cargo (including proteins and nucleic acids), ranging from 30 nm to 1000 nm in diameter, and their characters have been highly conserved throughout evolution. EVs have targeting abilities and the potential to transmit multidimensional, abundant and complicated information, as powerful and substantial “dogrobbers” mediating intercellular communications. As research has progressed, EVs have gradually become thought of as “dogrobbers” in bone tissue—the “eternal battle field” —in a delicate dynamic balance of destruction and reconstruction. In the current review, we give a brief description of the major constituent cells in bone tissues and explore the progress of current research on bone-derived EVs. In addition, this review also discusses in depth not only potential directions for future research to breakthrough in this area but also problems existing in current research that need to be solved for a better understanding of bone tissues.

## Background

Bone is a very well-adapted tissue in a delicate dynamic balance, hosting finely-tuned bone mineral metabolic processes for the homeostasis of both bone itself and other organs [1]. Emerging evidence has gradually shown that cells in bone are responsible for many effects on other systems, including the central nervous system (CNS), blood sugar, energetic metabolism and gonad function [2–4]. Moreover, other systems (including the endocrine system, CNS and digestive system) are also responsible for many effects on bone metabolism [5, 6]. Various kinds of growth factors and hormones impact the dynamic balance and homeostasis of bone tissue [7–9]. Osteocalcin (OCN), a specific protein secreted by osteoblasts (OBs), showed modulatory functions on gonad function and pancreatic insulin secretion [2–4].

**Fig. 1:**
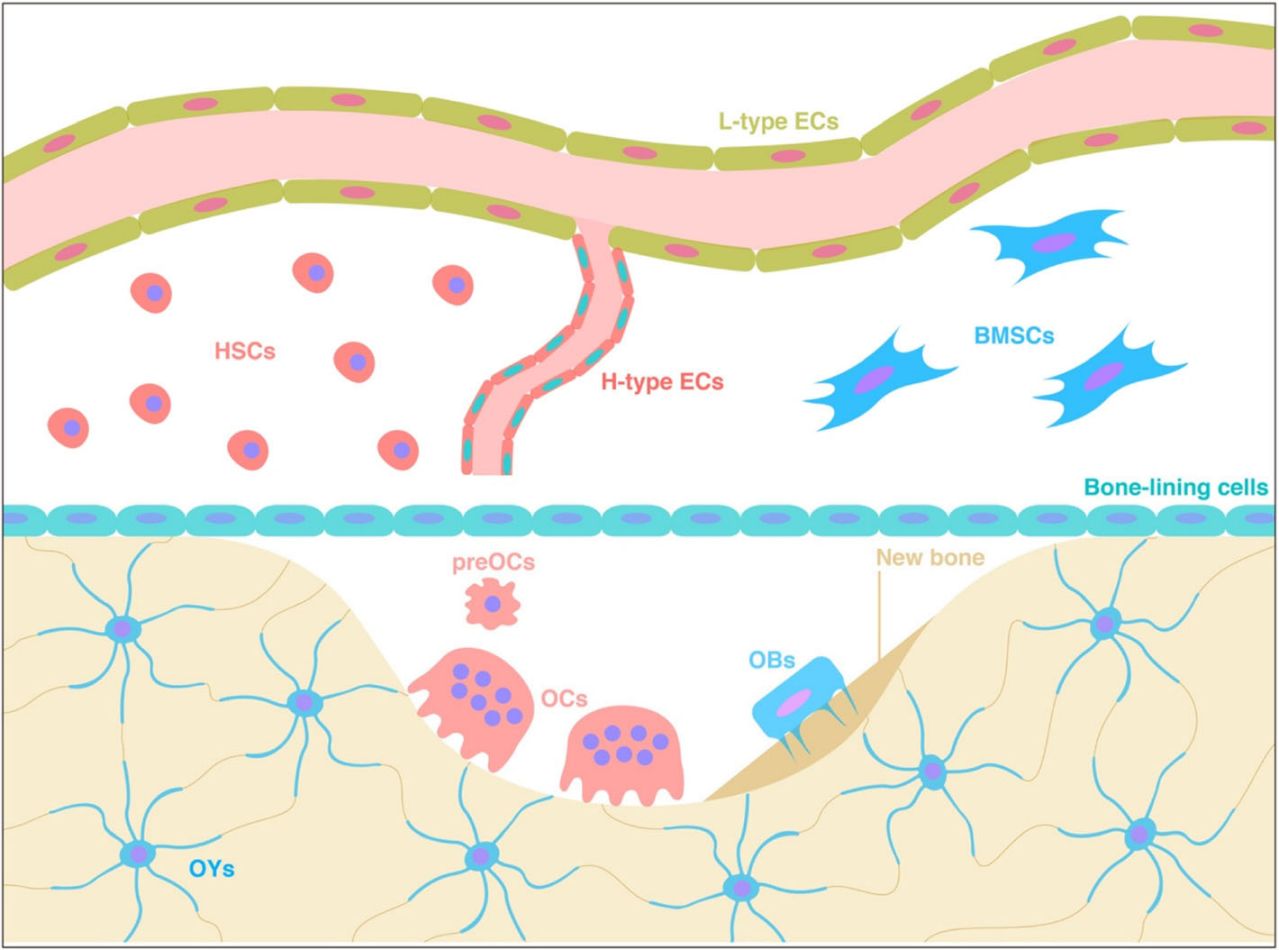
Bone remodelling compartment. OBs: osteogenic cells; OCs: osteoclasts; preOCs: pre-osteoclasts; OYs: osteocytes; HSCs: haematopoietic stem cells; BMSCs: bone mesenchymal stem cells; H-type ECs: endothelial cells strongly expressing both CD31 and endomucin (Emcn); L-type ECs: endothelial cells strongly expressing Emcn but not CD31

In the bone remodelling compartment (BRC), remodelling takes place throughout the whole life of organisms, including mammals, to maintain bone homeostasis via the replacement of senescent bone and repair of micro-damage [10]. The normal status of this remodelling is essential for the maintenance of both bone mass and bone mechanical properties [11]. In pathological conditions or with ageing, bone resorption exceeds bone formation, leading to low bone mass (osteopenia) or more seriously, osteoporosis [12]. In the opposite situation, bone formation can exceed bone resorption, leading to abnormally high bone mass, or osteosclerosis [12]. During the process of remodelling, a collaboration or symphony exists between two cell types with opposite functions—bone resorption by osteoclasts (OCs) and bone synthesis by OBs [13]. In addition to the two types of precursor cells, osteocytes (OYs) and vascular endothelial cells (ECs) are also responsible for regulating the balance of bone resorption and formation (Fig. 1) [10].

However, identification of the specific mechanisms and agents that are responsible for directly triggering and coordinating the processes of remodelling remains difficult. Considering the elaborateness of the regulation of bone remodelling, some communication methods among bone cells are needed to carry abundant information and help coordinate the regulatory processes.

Extracellular vesicles (EVs) have complex membrane structures and range from 30 nm to 1000 nm in diameter [14–17]. Almost all kinds of cells can secrete EVs, and the process has been highly conserved throughout evolution across many organisms from bacteria to plants and mammals [18–20]. In the human body, EVs can be isolated from almost every kind of biological body fluid, including saliva, plasma, ascitic fluid, amniotic fluid, breast milk, and urine [21–23].

**Fig. 2:**
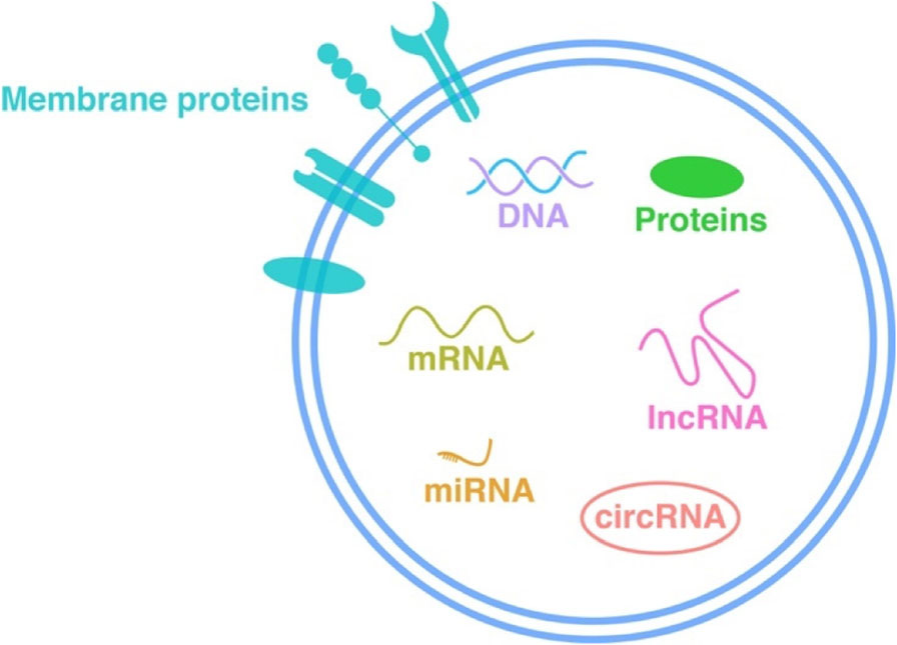
Constituents of EVs. EVs are composed of lipid bilayers, membrane proteins, intravesicular proteins, DNA, messenger RNAs (mRNAs), microRNAs (miRNAs), long noncoding RNAs (lncRNAs), and circular RNAs (circRNAs). The lipid bilayers provide protection from the complex and potentially harmful body fluid environment to the bioactive substances within EVs, the membrane proteins give EVs targeting abilities, and the various intravesicular contents give EVs the ability to transmit multidimensional, abundant and complicated information

EVs are composed of lipid bilayers, membrane proteins and intravesicular cargo (including proteins and nucleic acids) [15, 24]. The lipid bilayers provide protection from the complex and potentially-harmful body fluid environment to the bioactive substances within EVs, the membrane proteins give EVs targeting abilities, and the various intravesicular contents (proteins, messenger RNAs [mRNAs], microRNAs [miRNAs], long noncoding RNAs [lncRNAs], circular RNAs [circRNAs], etc.) give EVs the ability to transmit multidimensional, abundant and complicated information [15, 24]; thus, EVs are powerful and substantial “liaison officers” or “dogrobbers” mediating intercellular communications (Fig. 2).

Classification of EVs is based on their size and mechanisms of biogenesis [24]. EVs can be simply divided into two main classes: exosomes and microvesicles [14]. Exosomes (ranging from 30 to 100 nm in diameter) originate from multi-vesicular bodies (MVBs) via “inward budding” and are released after fusion between MVBs and the cytoplasmic membrane [14, 15]. Microvesicles (ranging from 100 to 1000 nm in diameter) originate and are released simultaneously at specific sites on the cytoplasmic membrane, named “microdomains”, via “outward budding” from the cytoplasmic membrane [14, 15]. In addition to the main classes, apoptotic bodies (ranging from 800 to 5000 nm in diameter) are vesicles that are shed during the apoptotic process [15]. However, apoptotic bodies rarely participate in intercellular communications, and one potential hypothesis suggests that they are eliminated by phagocytes immediately after their release [15].

Leading researchers in the EV research area, such as Clotilde Thery, suggested that although exosomes and microvesicles have different mechanisms of biogenesis, the present isolation technology is not capable of separating the sub-populations of EVs [25]. The term exosomes in most studies has been used to refer to a mixed sub-population of small EVs, so these researchers have suggested using a more generic term—EVs—when discussing the biological functions but not the mechanisms of biogenesis [25]. Hence, in the later sections of our review, we have used ‘EVs’ instead of ‘exosomes’ or ‘microvesicles’.

The continuous evolution of EV research may be essential for understanding the underlying mechanisms of communications not only among bone cells but also between bone and other body systems. Finally, our understanding of physiology and pathology may be dramatically enhanced. In this review, we introduce the major constituent cells in bone tissues, present the current research progress on EVs derived from these kinds of bone cells, and then point out the problems existing in current research with possible future research directions to achieve a breakthrough in this area.

## Cells in bone

Bone is constantly in a delicate dynamic equilibrium of both synthesis and resorption throughout the lives of animals including humans to withstand the demands of their activities. In the process of bone modelling and remodelling, bone-building cells (OBs) are responsible for osteogenesis (or bone synthesis), while bone-resorbing cells (OCs) are responsible for osteolysis (or bone resorption) [13]. In addition to the two ‘executors’, there are several kinds of cells proven to be responsible for the equilibrium of bone mineral metabolism as ‘supervisors’ or ‘regulators’. We will first introduce the background information about the major players in bone modelling and remodelling in order to summarize EVs as intracellular communicators among these cell types.

### OBs

OBs make up approximately 5% of the total resident cells in bones [26]. OBs are derived from bone mesenchymal stem cells (BMSCs) [27] and produce bone extracellular matrix (ECM) containing specific proteins, including OCN, alkaline phosphatase (ALP) and type I collagen (Col-I) [28]. In the osteogenic process, the crucial signalling pathways contain bone morphogenetic proteins (BMPs), Wnt proteins, ATF4, Runx2 and Osterix [26, 27, 29–33].

Theoretically speaking, OBs can be classified as preosteoblasts, mature OBs, and bone-lining cells [28]. This classification is based on in vivo histological analysis. However, the problem with this classification is the lack of a definite boundary between these subclasses [28]. Although there are methods for isolating OBs and cell lines that are usually used to study the function of OBs in vitro [34, 35], strictly defined mature OBs should be post mitosis, remain stationary [28] and can be very difficult to culture in vitro. Although in vitro cultured OBs are not strictly defined OBs, they perform well as a good cell model to study the functions of broadly defined OBs in vitro. Some recent viewpoints suggest giving these cells, which have similar functions and are responsible for osteogenesis, a unified name—osteogenic cells [36]. In this review, we use the more widely used term ‘OBs’ to refer to broadly defined OBs or osteogenic cells.

### OCs and preOCs

OCs are derived from haematopoietic stem cells (HSCs) [37]. In the process of osteoclastogenesis, the pre-osteoclasts (preOCs), which are tartrate-resistant acid phosphatase–positive (TRAP+) mononuclear cells, are formed first [38]. Then, preOCs form OCs, the TRAP+ multi-nuclear cells, via fusion [38, 39]. The functional disruption of OCs—the only known type of cell that is responsible for the resorption of bone matrix—is the key to pathogenesis related to bone metabolism. For instance, excessively activated OCs will cause osteoporosis and bone destruction [40]. In contrast, the impairment of OC formation or function will cause osteopetrosis [41–43].

According to current research, the most important signalling pathway regulating the differentiation of OCs is the receptor activator of nuclear factor kappa-B ligand (RANKL) signalling pathway. After receptor activator of nuclear factor kappa-B (RANK) is bound by RANKL, tumour necrosis factor receptor-associated factor 6 (TRAF6) is recruited to activate nuclear factor kappa-B (NF-κB) and the downstream cascades [44, 45]. Then, after NF-κB translocates into the nucleus, the transcription of OC-specific genes is initiated [44, 45]. In addition, mitogen-associated protein kinase (MAPK) signalling pathways, including p38, extracellular signal-regulated kinase (Erk), Jun N-terminal kinase (JNK), c-Fos and AP-1, are also essential for the formation of OCs [46–49].

Furthermore, cross-talk between signalling pathways is also important in osteoclastogenesis, such as partial calcium-signalling pathway activation by RANKL [50, 51] and nuclear factor of activated T-cells 1 (NFATc1), which are key during terminal OC differentiation and are common downstream of NF-κB, MAPK, and calcium-signalling pathways [52]. In contrast, the essential modulator responsible for cellular and systemic energy homeostasis, the AMP-activated protein kinase (AMPK) pathway, works as a suppressor of OC formation [53].

For a long time, PreOCs were considered to be simply precursors of OCs [54]. However, the true purpose will always be revealed. Interestingly, an increasing number of studies implied that preOCs may play a role in bone mineral metabolism that is completely different from that of OCs [55]. A large number of preOCs were found in regions associated with rapid growth of bone tissues, such as the periosteal bone surface [54–56]. In a more in-depth study using v-ATPase V0 subunit d2-deficient mice, Lee et al. blocked the fusion process of preOCs, leading to a deficiency of OC maturation, and then stimulated bone formation [40]. Xie et al. reported that preOCs promoted both angiogenesis and osteogenesis by targeting H-type vessels (or vessels expressing high levels of both CD31 & endomucin [Emcn]), and the potential key factor may be platelet-derived growth factor-BB (PDGF-BB) [55].

Some signalling pathways of preOCs have been discussed above. In addition, macrophage colony-stimulating factor (M-CSF) plays an important role in triggering the proliferation and differentiation of preOCs via activation of MAPK-Erk and phosphoinositide 3-kinase (PI3K)/protein kinase B (PKB; also known as Akt) signalling [57]. However, the research on preOCs is in its relative infancy with strong potential as not only a therapeutic target but also a candidate for cell therapy or EV-mediated non-cell therapy and thus needs further investigation.

### OYs

OYs, which are terminally-differentiated from OBs, are the most abundant sub-population of bone cells at over 90% [58–60]. OYs, which measure 9 μm by 20 μm in *Homo sapiens*, are star-shaped OB-derived cells embedded in mineralized bone matrix that reside in the lacunae as an interconnected network and are bathed in the canalicular fluid [61–63]. The highly-organized three-dimensional network of OYs gives them an enhanced adaptive ability [64]. Furthermore, the unique canalicular fluid is one of the information exchange hubs between OYs and the extracellular space containing important substances, including oxygen, nutrients, hormones and growth factors [63]. In the past, OYs were once considered quiescent within the mineralized matrix of bone to observational researchers [61]. As research technologies for studying OYs have advanced, the crucial role of OYs in the regulation of bone mineral metabolism has been gradually revealed.

There are two fundamental and well-recognized concepts in the research area of bone biology [65–69]: (1) Remodelling is primarily ‘damage-driven’, including ‘micro-damage’; (2) the processes of ‘damage-driven’ remodelling are mainly orchestrated by OYs.

The dendritic processes of OYs, which extend through the canaliculi, are essential for forming a highly-connected network not only between OYs but also between OYs and their neighbours, including OBs, OCs and cells in the blood supply in bone [70, 71]. OYs are generally believed to be important mechanosensors of bone tissue and are able to transduce mechanical signals into chemical signals after mechanical stimuli [70, 72]. OYs are also responsible for directly sensing ‘damage’ that interrupts the canaliculi [73–75].

Several signalling pathways are involved in OY-predominated regulation and control of OBs and OCs. These pathways are the major sources of sclerostin, the primary candidate for regulating OBs [59, 76, 77], and RANKL, the key modulatory factor of bone resorption via regulating OCs [78]. These two proteins are the major signals involved in bone metabolism [79–81].

**Fig. 3:**
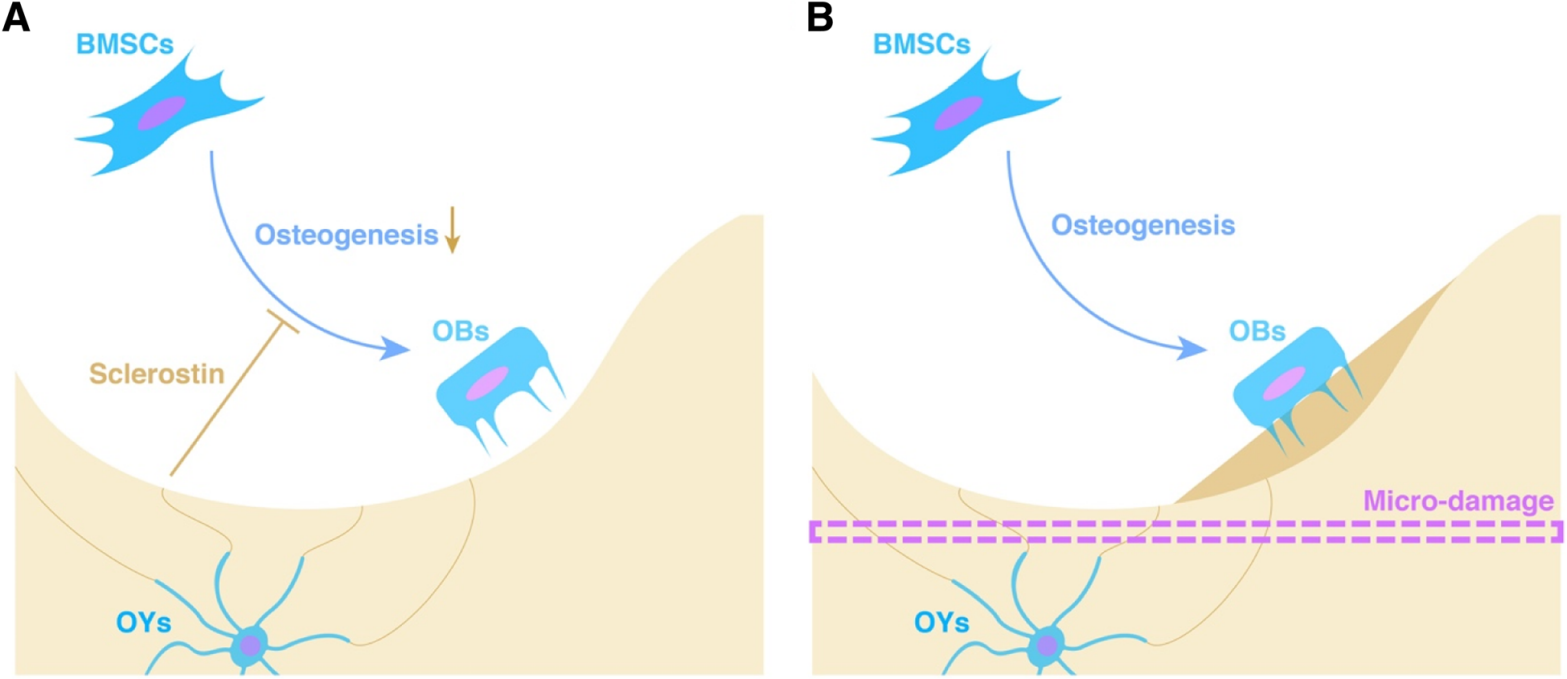
‘Micro-damage-driven’ remodelling orchestrated by OYs via sclerostin. OYs are responsible for directly sensing ‘damage’ that interrupts the canaliculi. Sclerostin has been reported to maintain the status ‘inhibition of OB differentiation’. However, mechanical loading or ‘damage’ would shut down sclerostin, causing a loss of efficacy of the ‘inhibition of OB differentiation’ function; in other words, increasing OB differentiation and bone mass

Sclerostin has been reported to inhibit the Wingless-related integration site (Wnt) pathway and then keep the status ‘inhibition of OB differentiation’ (Fig. 3). However, mechanical loading or ‘damage’ would shut down sclerostin, causing a loss of efficacy of the ‘inhibition of OB differentiation’ function, in other words, increasing OB differentiation and bone mass [82–85]. Interestingly, mechanical unloading causes high sclerostin levels together with profound bone loss, and this phenomenon might be corroborative evidence of sclerostin’s function in OY-mediated bone mass modulation [86]. RANKL derived from OYs participates in osteoclastogenesis and OC activity and then modulates the resorption of bone tissue [87].

Thus, OYs have the potential to be an orchestrator of mineral metabolism and bone remodelling [61, 88] and may be potential targets of osteoporosis treatment [89, 90]. However, there are many mysteries about how OYs transmit key molecules to target cells.

In addition, recent evidence implied that OYs regulate not only bone but also systemic processes of multiple organs. Sato et al. reported that ablation of OYs in mice (OY-less mice) leads to both severe lymphopenia and the loss of white adipose tissue [91]. In a separate study, Sato and his colleagues found that the long distance and systemic functions may be due to EVs secreted by OYs [92].

### ECs

In the beginning, the endothelium was once considered to be just a ‘cellophane wrapper’ providing selective permeability to electrolytes and water, without any further function. As research progressed, more and more evidence has led to a more in-depth understanding of the multitudinous functions of the endothelium [93, 94]. The basic unit of the endothelium is the EC. These cells have very special properties and functions which dominate vascular biology, including fluid filtration, blood flow regulation, interaction with platelets and leukocytes, and very importantly, angiogenesis. [94–96].

The coupling of angiogenesis and osteogenesis plays an essential role in maintaining appropriate bone homeostasis [97, 98]. The specific performance is the result of the coincidence between vascular invasion and the appearance of OBs and OCs [97, 99]. There are highly branched, irregular, and discontinuous networks known as bone marrow capillaries, in addition to feeding arteries and draining veins [100, 101]. Recently, bone marrow capillaries have been classified as two specific subtypes—H-type vessels (or vessels strongly expressing both CD31 & Emcn) and L-type vessels [99].

Specifically, the H-type vessel may be the key to coupling, while the L-type vessel (hierarchically downstream of H-type vessels) is not associated with promoting osteogenesis and coupling [99, 102]. In addition, the ECs of H-type vessels (H-type ECs), which are found at the front of the vascular growth direction in bone tissue and express high levels of Emcn and CD31, are key in promoting osteogenesis [99, 102].

In mouse models, there is a very interesting phenomenon in which the total number of ECs in bone is not significantly different after maturation but the ratio of H-type:L-type ECs decreases with age after adolescence, and this phenomenon implies that the relationship between the transformation from H-type ECs to L-type ECs and the bone mass loss during ageing is caused by a loss of osteogenic ability [99]. Furthermore, the Notch and hypoxia-inducible factor (HIF) signalling pathways have been identified as essential signalling pathways that promote H-type vessel expansion together with the associated osteogenesis [99, 102].

Current research shows that there are two main communication approaches: a direct action and a remote action. At present, a direct action approach has only been found between ECs and BMSCs. EC-derived PDGF-BB recruits PDGF-receptor β (Rβ)–expressing BMSCs into a pericyte fate [103]. Recruited BMSCs (or pericytes), which wrap around a blood vessel like a stocking, express angiopoietin-1, which signals via Tie-2 expressed in ECs, and is essential for the integrity and stabilization of blood vessels [104]. For a remote action, early studies have mainly focussed on protein factors. ECs produce several factors that support HSCs including chemokine CXC ligand 12 (CXCL12), angiopoietin and stem cell factor (SCF) [105]. ECs also produce factors including BMPs, and have direct effects on OBs [106]. Besides, there are studies reporting that EC-derived netrin-4 participates in the regulation of OCs to maintain bone stabilization [107]. With advances in this area, EVs derived from ECs have gradually received more attention, which will be introduced later. Nonetheless, some confusing issues remain regarding how ECs communicate with other cells in bone and cooperate with each other for bone homeostasis. In future studies, there are several questions that must be answered: 1) whether there are more direct interactions between ECs and other bone cells; 2) what other important protein factors are involved; 3) the function and mechanism of EVs derived from ECs; 4) what differences exist between H-type ECs and L-type ECs in communication with other bone cells; 5) whether and how H-type ECs and L-type ECs communicate with each other.

### BMSCs

In the bone marrow, BMSCs (also called bone marrow stromal cells) are mononuclear stem cells that are osteogenic precursors and can differentiate into OBs and other kinds of skeletal cells [108, 109]. There is a widely-used method to isolate BMSCs. After bone marrow cells are seeded into culture flasks, the cell population rapidly grows, with cells having a spindle-shaped fibroblast-like morphology and the potential to differentiate into osteocytes, adipocytes and chondrocytes, and this multilineage potential lasts for several passages. The multipotential cells isolated using this method are usually called ‘BMSCs’ [110, 111].

However, after years of research, some problems have received increasing attention. The in vivo BMSCs have been reported to be very different from ex vivo expanded ‘BMSCs’ [111, 112], and even multilineage potential stem cells similar to these ex vivo ‘BMSCs’ can be isolated from several tissues other than bone marrow [113, 114]. Thus, the so-called ex vivo expanded ‘BMSCs’ are best identified as skeletal stem cells (SSCs), which lack markers to identify them in situ [111, 112]. Unfortunately, these problems might mean that we have little or almost no knowledge of true in vivo BMSCs, which we had thought was very clear.

Nevertheless, optimistically speaking, there have been many achievements. In the field of application, SSCs (or what we usually called ‘BMSCs’) are very good seed cells for tissue repair and one of the representative cells used to observe the response of bone cells after repeated stimulation. The gaps in in vivo BMSC knowledge are tremendous opportunities for future research to better understand the fascinating mysteries of biology.

## EVs in bone

### OB-EVs

Proteins and nucleic acids, contained within OB-derived EVs (OB-EVs), play important roles in intercellular communication in bone tissue [115]. OB-EVs were once called matrix vesicles several years ago and were considered to be OB-originated EVs only recently [116–118].

OB-EVs contain RANKL, which can be transferred to precursors of OCs. Via receptor–ligand interaction, OB-EVs can stimulate RANKL–RANK signalling to promote osteoclast formation [119]. Inhibition of OB-EV production can prevent bone loss [120]. These results mean that OBs can stimulate the formation and activation of OCs via EVs without direct cell-to-cell contact. Interestingly, parathyroid hormone (PTH) can induce an increase in RANKL expression in OB-EVs [119]. This finding may imply that OB-EVs are participants in the regulation of hormone-related bone remodelling.

In addition to RANKL, galectin-3 has also been found to be important in OB-EVs. Weilner et al. reported that EVs from elderly people can inhibit the osteogenic ability of BMSCs unlike those from younger people [121]. Interestingly, the galectin-3 content was decreased in EVs from elderly people [121]. Through several in vitro assays, Weilner et al. proved that the intravesicular galectin-3 levels are positively correlated with osteogenic potential and are responsible for the biological responses of BMSCs to OB-EVs [121].

Recently, Ge et al. reported the results of a proteomics study on EVs derived from MC3T3 cells (mouse OBs) and showed that many proteins, which were highly enriched in EVs derived from MC3T3 cells, are enriched in osteogenesis-related pathways, including eukaryotic initiation factor 2 (EIF2) signalling, integrin signalling and mammalian target of rapamycin (mTOR) signalling [122]. EIF2 signalling participates in bone morphogenetic protein 2 (BMP2)-induced OB differentiation [123]. Integrin signalling promotes angiogenesis and thus participates in bone formation, remodelling, and fracture healing [124]. Furthermore, the activation of mTOR signalling can increase osteogenesis via the suppression of peroxisome proliferator-activated receptor-γ (PPARγ) [125]. However, proteomics studies are just for preliminary screening of key molecules, and more in-depth studies are needed in the future.

OB-EVs also contained microRNAs (miRNAs). Cui et al. found that several miRNAs in OB-EVs were increased during mineralization [126]. Through co-target analysis and in vitro assays, they found that five upregulated miRNAs (miR-667-3p, miR-6769b-5p, miR-7044-5p, miR-7668-3p and miR-874-3p) can co-target a negative regulator of the Wnt signalling pathway—Axin1—and then enhance the expression level of β-catenin [126].

### OC-EVs and PreOC-EVs

**Fig. 4:**
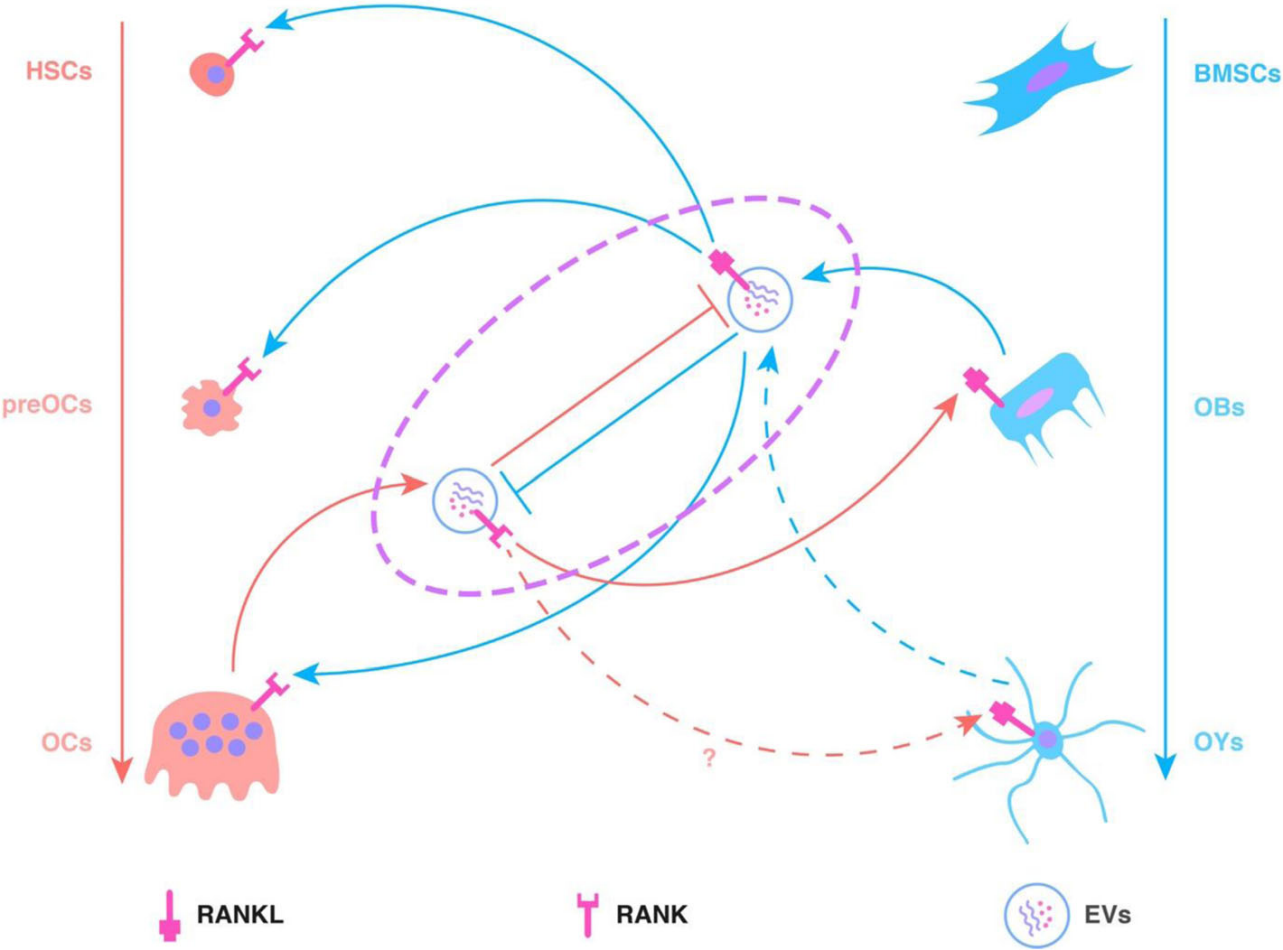
RANK–RANKL interaction mediated by EVs. RANK-containing OC-EVs target OBs via the RANK–RANKL interaction. RANKL-containing OB-EVs target HSCs/preOCs/OCs via the RANK–RANKL interaction. In addition, RANK-containing OC-EVs and RANKL-containing OB-EVs can block the functions of each other

Proteins and nucleic acids in EVs derived from OCs (OC-EVs) may also play an important role in bone mineral metabolism. Huynh et al. found that OC-EVs, which contain RANK on the surface, are paracrine regulators of osteoclastogenesis [127]. RANK-containing OC-EVs may target OBs via the RANK–RANKL interaction, and RANKL-containing OB-EVs may also target OCs via the RANK–RANKL interaction. RANK/RANKL may give EVs a natural targeting ability to transfer other proteins and nucleic acids and contribute to the reprogramming of target cells. In addition, RANK-containing OC-EVs may block the function of RANKL-containing OB-EVs, thus inhibiting the formation of OCs (Fig. 4) [127].

Increased levels of serum miR-214-3p contained within EVs were found in both elderly women with fractures and ovariectomized (OVX) mice [128]. Li et al. observed that OCs are the primary source of EVs containing miR-214-3p. Hence, they employed an OC-specific miR-214-3p knock-in mouse model and found that the serum level of miR-214-3p contained within EVs increased while bone formation decreased, but could be rescued by using the OC-targeted RNA antagonist antagomir-214-3p [128]. Sun et al. found similar results showing that miR-214 is responsible for the inhibition of osteogenesis by targeting OBs. With more in-depth studies, they also found that OC-EVs specifically recognized OBs through the interaction between ephrin-A2 (carried by OC-EVs) and EphA2 (on OBs) [129]. miR-214 directly targets activating transcription factor 4 (ATF4) to inhibit the activity of OBs [130]. These results implied that OCs communicate with OBs via EVs to inhibit osteogenic ability. Furthermore, miR-214 in EVs could be a possible diagnostic, prognostic and therapeutic target for osteoporosis.

Huynh et al. found RANK in OC-EVs but not in EVs derived from preOCs (preOC-EVs) [127]. More interestingly, preOC-EVs can promote the formation of OCs, but OC-EVs can inhibit the formation of OCs [127]. This phenomenon is very similar to positive and negative feedback and may be essential for the stabilization of bone mineral metabolism. Hence, it is possible that the constitution and function of preOC-EVs might be completely different from those of OC-EVs, consistent with the functional differences between preOCs and OCs. However, research is still lacking in this area, and further research is needed.

### OY-EVs

As briefly described in a previous section, Sato and his colleagues found that EVs derived from OYs (OY-EVs) may transfer their components, including miRNAs, to recipient cells via circulation in the blood [92]. This finding might be the key to solving the puzzle of the mechanisms underlying the regional and systemic functions of OYs.

Morrell et al. found that mechanical stimulation upregulates the production of EVs containing bone regulatory proteins, coupled with Ca^2+^-dependent contractions [131]. miR-218, contained within OY-EVs, inhibits sclerostin and influences the differentiation of OBs [132]. Interestingly, the OY-derived miR-218 contained in OY-EVs can be suppressed by myostatin, which is secreted by muscles [132]. These results provide potential mechanisms for muscle–bone communications and may be one of the potential hypotheses explaining why muscle activity is essential for maintaining normal bone density. However, this research is still in its infancy. RANKL and sclerostin are key molecules derived from OYs, but there is no evidence about whether they are found in OY-EVs. Hence, there is an urgent need for proteomics and transcriptomics studies to reveal the core of OY-EVs.

### EC-EVs

miR-31 is highly expressed in EC-derived EVs (EC-EVs) from elderly and osteoporotic patients [133]. miR-31 contained within EVs derived from senescent ECs was taken up by BMSCs and targeted Frizzled-3 to inhibit osteogenic ability [133]. miR-31 from within the senescent EC-EVs might be a potential valuable biomarker and therapeutic target of osteoporosis. Nonetheless, there is still not enough research. It would be better to execute a specific study about H-type EC-EVs rather than general EC-EVs.

### BMSC-EVs

EVs derived from BMSCs (BMSC-EVs) are also important participants in the remodelling of bone tissue via direct regulation of the proliferation and activity of OBs [134]. Davis et al. found that oxidative stress could elevate miR-183 levels in BMSC-derived EVs (BMSC-EVs), which could induce senescence [135]. Recently, Xu et al. [136] observed that in aged rats, which exhibited increased adipogenesis and decreased osteogenesis, miR-31a-5p was significantly increased in BMSCs. Furthermore, they found that miR-31a-5p was also significantly increased in BMSC-EVs and that miR-31a-5p in BMSC-EVs was responsible for the activated osteoclastogenesis in aged rats. Thus, age-related miR-31a-5p in BMSCs could be a potential therapeutic target due to its dual functions as an inhibitor of osteogenesis and activator of osteoclastogenesis.

However, most recent studies related to BMSC-EVs have concentrated on therapeutic use under a variety of conditions, including anti-inflammation, tissue repair and tissue protection. Another focus of BMSC-EV research is the relationship between BMSC-EVs and tumours. Nevertheless, the in-depth study of BMSC-EVs in bone cell communications under physiological and pathogenic conditions is still relatively insufficient. Certainly, BMSC-EVs have a powerful potential as therapeutic drugs for regenerative medicine or targets of tumour therapy, but revealing the specific details of BMSC-EVs in the communication of other bone tissue cells is important.

Recently, the so-called ex vivo expanded ‘BMSCs’ have been identified as SSCs [111, 112]. Optimistically speaking, the massive amount of research on the therapeutic use of EVs derived from SSCs (or what we usually call ‘BMSCs’) will receive very little attention (at most, the name might change). These results also mean that we have virtually no knowledge about the in vivo function of BMSC-EVs. In other words, the lack of in vivo BMSC-EV knowledge may be a tremendous opportunity for future studies to better understand the intercellular communication among bone cells.

## Discussion and outlook

With the development of research, EVs have gradually come to be thought of as correspondents or “dogrobbers” in bone tissue, the “eternal battle field” in a delicate dynamic balance of destruction and reconstruction. An in-depth understanding of the EVs in bone helps to understand both how bone maintains a delicate homeostatic balance and the pathogenesis of disease.

Furthermore, there is another mystery—how bone communicates with other systems. The conventional hypothesis is that growth factors, hormones and specific proteins such as OCN participate in the communication between bone and other systems [2–4, 7–9]. However, growth factors, hormones and specific proteins (such as Wnt) were also found to be carried by EVs in many studies [137–140]. Relative to the free state, EVs provide protection for bioactive macromolecules from a complex humoral environment [15, 141, 142]. According to this evidence, EVs may also be the “trucks” used by OCN to make it possible to influence other organs/systems, but this hypothesis needs confirmation from future studies. Several studies have shown that OCN can be carried by EVs [143, 144]. Soriano et al. found that EVs may participate directly in the process of vascular calcification via OCN [144]. However, at present, there is still too little relevant research, and current research is also not sufficiently in depth. Nevertheless, this situation means that EVs carrying bone-specific contents and their functions in other organs will be very promising future research areas. Furthermore, EVs can carry much more complex information than hormones and growth factors [145–147], and they have certain targeting properties to precisely regulate specific organs/systems [15, 148]. Therefore, future research can focus on EVs, and a breakthrough in this field is most likely.

Due to the limitations of current research techniques, the functional similarities and differences among sub-populations of EVs cannot be explained clearly. Moreover, many studies do not make clear distinctions. We have used the term ‘EVs’ rather than the terms of their sub-populations in this review. However, the sub-populations of EVs is a topic attracting considerable interest, and increasing attention has been paid to it [149, 150]. Increasing numbers of researchers will be involved in this area, and the understanding of both bones and the function of their EVs will be more in depth.

OBs and OCs can exist within the periosteum and the bone marrow. There are obvious differences between these two sites. OBs/OCs in the periosteum have more possibilities to communicate with tendons, muscles and nerves. In addition, OCs in the periosteum are derived from circulating monocytes [151], while OCs in the bone marrow are derived from HSCs. Although the precursors of both are the same—HSCs—whether the two have some differences has yet to be determined. In current research, there is no good distinction in the sub-population of OBs. In particular, there is a lack of in vitro research tools for studying specific subtypes of OBs in different stages, and progress in this area will bring about profound changes.

OYs, the most abundant sub-population of bone cells, are the least-studied cell type. Current research suggests that sclerostin is a very important molecule. Micro-damage causes sclerostin shut-down and triggers bone remodelling. However, it is not clear whether sclerostin is present in a free state or in EVs. We suspect that sclerostin exists in EVs and that the EVs are present in canalicular fluid. Moreover, micro-damage may block the mobility of canalicular fluid and then cause a shut-down of EV-carried sclerostin. Follow-up studies should first confirm what is affected by the micro-damage—the OYs, the mobility of canalicular fluid, or both.

There are many studies on EC-derived EVs. However, current studies have not focused on H-type ECs. Not only are there few studies on H-type ECs, but there is also almost no research on EVs derived from H-type ECs. Future research in this direction is likely to be one of the most important advances. Beyond that topic, there are large gaps with enormous research potential regarding the true nature of BMSCs, as mentioned in the previous section.

EVs have gradually become a focus of the new generation of treatment and diagnosis. With all kinds of new technologies [152], we can likely use non-invasive and convenient methods to monitor the status of bone and prevent many joint system-related diseases, and ultimately to improve human health with a high quality of life.

## Conclusions

Bone is a very well-adapted tissue in a delicate dynamic balance, hosting finely-tuned bone mineral metabolic processes for bone homeostasis by collaboration or symphony among several cell types including OCs, OBs, OYs, ECs and their precursors. EVs are widely-distributed vesicles, ranging from 30 nm to 1000 nm in diameter, with targeting abilities and potential to transmit multidimensional, abundant and complicated information. In this review, we introduce the major constituent cells of bone tissues and explore the progress of current research into bone-derived EVs and then point out the problems existing in current research with possible future research directions to break through in this area.

## Abbreviations

ALP: Alkaline phosphatase
AMPK: AMP-activated protein kinase
ATF4: Activating transcription factor 4
BMP2: Bone morphogenetic protein 2
BMPs: Bone morphogenetic proteins
BMSC-EVs: EVs derived from BMSCs
BMSCs: Bone mesenchymal stem cells
BRC: Bone remodelling compartment
circRNAs: Circular RNAs
CNS: Central nervous system
Col-I: Type I collagen
CXCL12: Chemokine CXC ligand 12
EC-EVs: EC-derived EVs
ECM: Extracellular matrix
ECs: Vascular endothelial cells
EIF2: Eukaryotic initiation factor 2
Emcn: Endomucin
Erk: Extracellular signal-regulated kinase
EVs: Extracellular vesicles
HIF: Hypoxia-inducible factor
HSCs: Haematopoietic stem cells
JNK: Jun N-terminal kinase
lncRNAs: Long noncoding RNAs
MAPK: Mitogen-associated protein kinase
M-CSF: Macrophage colony-stimulating factor
miRNAs: MicroRNAs
mRNAs: Messenger RNAs
mTOR: Mammalian target of rapamycin
MVBs: Multi-vesicular bodies
NFATc1: Nuclear factor of activated T-cells 1
NF-κB: Nuclear factor kappa-B
OB-EVs: OB-derived EVs
OBs: Osteoblasts
OC-EVs: EVs derived from OCs
OCN: Osteocalcin
OCs: Osteoclasts
OVX: Ovariectomized
OY-EVs: EVs derived from OYs
OYs: Osteocytes
PDGF-BB: Platelet-derived growth factor-BB
PDGF-Rβ: Platelet-derived growth factor-receptor β
PI3K: Phosphoinositide 3-kinase
PKB: Protein kinase B
PPARγ: Peroxisome proliferator-activated receptor-γ
preOC-EVs: EVs derived from preOCs
preOCs: Pre-osteoclasts
PTH: Parathyroid hormone
RANK: Receptor activator of nuclear factor kappa-B
RANKL: Receptor activator of nuclear factor kappa-B ligand
SCF: Stem cell factor
SSCs: Skeletal stem cells
TRAF6: Tumour necrosis factor receptor-associated factor 6
TRAP+: Tartrate-resistant acid phosphatase-positive
Wnt: Wingless-related integration site
